# Central posterior hyaloid fibrosis: evolution and outcomes

**DOI:** 10.1186/s40942-023-00494-5

**Published:** 2023-09-07

**Authors:** Ramesh Venkatesh, Ashit Handa, Vishma Prabhu, Sai Prashanti Chitturi, Aishwarya Joshi, Isha Acharya, Rubble Mangla, Naresh Kumar Yadav, Jay Chhablani

**Affiliations:** 1 Dept. of Retina and Vitreous, Narayana Nethralaya, #121/C, 1st R Block, Chord Road, Rajaji Nagar, 560010 Bengaluru, Karnataka India; 2 Medical Retina and Vitreoretinal Surgery, University of Pittsburgh School of Medicine, 203 Lothrop Street, Suite 800, Pittsburg, PA 15213 USA

**Keywords:** Age related macular degeneration, Macular neovascularisation, Fibrosis, Inflammation, Central posterior hyaloid fibrosis

## Abstract

**Purpose:**

To report contributory factors and clinical outcomes of central posterior hyaloid fibrosis (CPHF) associated with neovascular age-related macular degeneration (nAMD).

**Methods:**

In this retrospective, single-center study, patients with CPHF and nAMD were included. Demographic and imaging characteristics, as well as the anatomical and functional outcomes, of these patients were analysed.

**Results:**

We identified 530 eyes in 273 patients with chronic predominantly scarred macular neovascularisation (MNV), and 32 eyes in 29 patients revealed CPHF, representing a prevalence of 6%. Patients had a mean age of 72.76 years. Before and during the development of CPHF, Type 2 MNV was observed in all eyes. At the time of MNV diagnosis, mean logMAR visual acuity was 1.308 ± 0.559 (20/407). The average time to develop CPHF was 27.3 months since the diagnosis of MNV. At the time of CPHF identification, the mean logMAR visual acuity was 1.498 ± 0.374 (20/630). RPE tear was observed in 6% (n = 2) of CPHF eyes. The average number of intravitreal anti-VEGF injections administered prior to the diagnosis of CPHF was 2.4 and after the onset of CPHF was 0.9. The final visual acuity was not significantly different at the final follow-up visit [1.304 ± 0.42 (20/402); p = 0.646].

**Conclusion:**

Rarely observed in eyes with predominantly scarred subfoveal type 2 MNVs and extensive subretinal fibrosis, CPHF is associated with poor visual outcomes. Its presence could possibly suggest a profibrotic effect of MNV on the posterior hyaloid. **Trial Registration Number:** Not applicable.

## Introduction

Recent years have witnessed the emergence of optical coherence tomography (OCT) as the investigation of choice for the diagnosis and management of neovascular age-related macular degeneration (nAMD) [1]. The literature has discussed a variety of OCT-based biomarkers that have demonstrated clinical utility in the diagnosis of nAMD. These biomarkers encompass the description and classification of macular neovascularization (MNV) characteristics, the evaluation of disease activity, the monitoring of treatment outcomes, and the prediction of visual prognosis [2]. MNV secondary to age-related macular degeneration is characterized by the aberrant development of blood vessels originating from the choroidal vasculature and extending into the neurosensory retina through Bruch’s membrane and the retinal pigment epithelium (RPE). Three distinct types of MNVs have been identified on OCT based on their location. Type 1 MNV originates from the choroid and is situated in the interstitial space between the RPE and the Bruch membrane. Type 2 MNV extends through the RPE and is located in the space between the RPE and the sensory retina. Type 3 MNV, which is also referred to as retinal angiomatous proliferation, manifests as the proliferation of abnormal blood vessels in the deep layers of the retina and choroid when it proliferates beneath the sensory retina and RPE [3, 4]. OCT has demonstrated its utility in the identification of vitreomacular changes in patients with nAMD. A number of studies have examined these modifications as a potential initiation point for the development of MNV [5, 6]. According to reports, the involvement of both vitreomacular adhesion (VMA) and vitreomacular traction (VMT) has been identified in the pathogenesis of MNV. On the other hand, it seems that posterior vitreous detachment may lead to a reduction in the occurrence of MNV [5]. The potential promotion of MNV by VMA may be attributed to several mechanisms. These include the induction of chronic low-grade inflammation, which facilitates the exposure of the macula to cytokines or free radicals present in the vitreous gel. Additionally, VMA may interfere with the trans-vitreous oxygenation and nutrition of the macula, further contributing to the development of MNV [7].

In our retina clinic, we noted a few cases of MNV secondary to nAMD that were subfoveal in location, predominantly scarred and showed fibrosis, with a dense pearly-white tissue firmly attached at its centre, with or without associated subretinal fluid (SRF) and/or intraretinal fluid (IRF) on clinical examination, and presented with a poor visual acuity. On OCT, these eyes demonstrated a specific vitreomacular interface change consisting of abnormal, focal hyperreflectivity of the posterior hyaloid membrane at the fovea, which appeared to connect with the subfoveal MNV located in the subretinal space. Such a similar clinical presentation and OCT description was noted in a recently published paper by Khan et al. [8] The novel OCT feature described by the authors is identified as “abnormal hyper reflectivity at the point of attachment of the posterior hyaloid membrane to the foveal center that appears to ‘fill in’ the foveolar depression without affecting foveolar contour (concavity) in the absence of wrinkling of inner retinal surface.“ The authors have named this feature central posterior hyaloid fibrosis (CPHF). The main objective of this study was to present this novel OCT finding and outline its distinctive features. However, this particular case series did not include an analysis of the predictive baseline characteristics of MNV that leads to CPHF. Additionally, there was a lack of information regarding the anatomical and visual outcomes in the eyes affected by this condition. It is of utmost importance to identify the fundamental predictive factors that may contribute to the emergence of this unfavourable prognostic OCT sign in the course of treating nAMD. This knowledge is essential for preventing the occurrence of CPHF and attaining a positive visual outcome in eyes affected by nAMD.

With this context in mind, we intended to investigate the factors that may have contributed to the development of this not so uncommon OCT finding in nAMD. In addition, we investigate the long-term clinical and functional outcomes of these eyes after the identification of CPHF.

## Methods

In this single-center retrospective study, 959 eyes of 488 patients grouped under the category of ‘MNV’ on the Spectralis OCT (Heidelberg Engineering, Germany) machine were screened to identify eyes with nAMD and predominantly scarred MNV and CPHF on OCT. This research was conducted at a tertiary eye hospital in South India between January 2011 and December 2022. The local Institutional Review Board/Ethics Committee approved the research because it adhered to the Declaration of Helsinki’s principles.

**Fig. 1:**
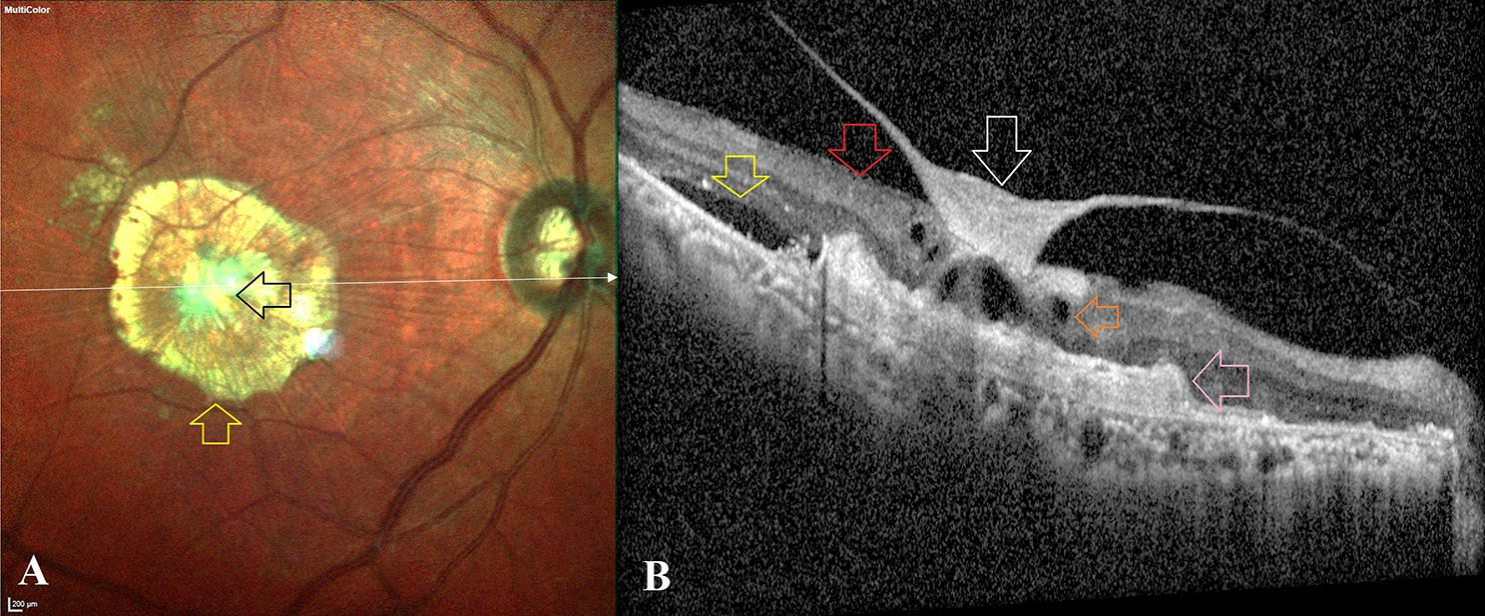
Descriptive features of central posterior hyaloid fibrosis: Fig. 1A: Clinical colour photograph using the Multicolour® imaging (Spectralis, Heidelberg Engineering, Germany) shows: a) Subfoveal, predominantly scarred macular neovascularization (MNV) (yellow arrow) with dense pearly-white tissue (black arrow) at its center. Figure 1B depicts the optical coherence tomography characteristics of CPHF acquired with the Spectralis machine. These includes: **(a)** abnormally hyperreflective posterior hyaloid membrane with vitreo-foveal adhesion (white arrow) with a filled-in appearance of the foveal depression; **(b)** absence of other vitreomacular interface abnormalities and inner retinal surface wrinkling (red arrow); (c) type 2 subfoveal MNV located in the subretinal space appearing predominantly scarred and fibrotic (pink arrow); (d) presence of subretinal fluid (yellow arrow) and (e) presence of intraretinal fluid (orange arrow)

MNV occupied with an area of scar tissue seen on clinical examination and characterized by hyperreflectivity from the MNV complex with underlying backscattering of light on OCT was considered as predominantly scarred MNV [1]. CPHF was defined as abnormal hyper reflectivity at the point of attachment of the posterior hyaloid membrane to the foveal center that appears to “fill in” the foveolar depression without altering foveolar contour (concavity) in the absence of inner retinal surface wrinkling **(**Fig. 1**)** in a predominantly scarred MNV eye. This CPHF description was drawn from a recent publication by Khan et al. [8].

The medical records of all patients with CPHF were reviewed, and the following information was collected: age, gender, involved eye, presence of associated medical conditions, best-corrected visual acuity in logarithm of minimum angle of resolution (logMAR) units prior to the identification of CPHF, at the time of identification of CPHF, and at the last follow-up visit. OCT features such as type of MNV, location of MNV in relation to the fovea, and presence of fluid in any of the three fluid compartments viz., sub RPE, sub retinal and intraretinal spaces, presence of associated signs of disease activity such as subretinal hyperreflective material (SHRM), hard exudates and/or hemorrhage, presence of RPE tear and treatment received by the patient before and after identification of CPHF. On OCT, RPE tears were identified by the absence of an RPE monolayer, an increased signal of the underlying choroid, and a free edge of a wavy, contracted RPE that was retracted towards the MNV [9]. In addition, the condition of the posterior hyaloid at the fovea and any associated epiretinal membrane was noted.

Treatment was administered in an effort to reduce disease activity (presence of subretinal or intraretinal fluid). Anti-vascular endothelial growth factor (VEGF) therapy was prescribed at the discretion of the clinician without adherence to any predetermined protocol. Aflibercept (Eylea®; Regeneron, Inc., Eastview, NY), bevacizumab (Avastin®; Genentech, Inc., South San Francisco, CA), innovator ranibizumab (Lucentis®; Novartis, USA), biosimilar ranibizumab (Razumab®, Intas Pharmaceuticals, India), and brolucizumab (Pagenax®, Novartis, India) were the anti-VEGF medications used. The demographic and imaging characteristics, as well as the anatomical and functional outcomes, of these patients with CPHF in nAMD were analysed further.

### Statistical analysis

All data were analysed using GraphPad Prism version 9.5.1 (733) for Windows, GraphPad Software, San Diego, California USA, www.graphpad.com. The Shapiro-Wilk normality test was used to test the normality of the data sets. The visual acuity was assessed using Snellen’s chart and then converted to logMAR units for statistical analysis. Comparison of changes in visual acuity at different time points in the study was done using Wilcoxon matched-pairs sign rank test. P values < 0.05 were considered statistically significant.

## Results

In this study, 530 eyes in 273 patients with predominantly scarred MNV and 32 eyes in 29 patients (6%) with CPHF on OCT were identified. This study included 20 (69%) males and 9 (31%) females. At the time of the onset of CPHF, the MNVs of all patients were predominantly scarred. At the onset of CPHF, the average age of patients was 72.76 ± 9.09 years. Eleven (34%) and 21 (66%) eyes were affected on the right and left, respectively. Table 1 explains the specific details:

**Table 1:** Demographic data of patients with central posterior hyaloid fibrosis:

Variable	Value
No. of patients/eyes screened with diagnosis of AMD MNV	359/715
No. of patients/eyes diagnosed with predominantly scarred AMD MNV (> 50% fibrosis)	273/530
No. of patients/eyes with CPHF	29/32
Males: Females	20:9
Laterality (RE: LE)	11:21
Mean age (years)	72.76 ± 9.09
Patients with history of diabetes mellitus (n, %)	15 (52)
Patients with history of hypertension (n, %)	12 (41)

Abbreviations: AMD – age related macular degeneration; MNV – macular neovascularization; CPHF – central posterior hyaloid fibrosis; RE – right eye; LE – left eye

CPHF was detected at presentation in 22 (69%) of eyes. In the remaining 10 eyes (31%), CPHF developed during subsequent visits. Table 2 lists the clinical and imaging characteristics of these 10 eyes prior to the identification of CPHF. Before the onset of CPHF, the mean logMAR visual acuity of these eyes was 1.308 ± 0.559 (20/407). Each eye had posterior hyaloid attachment at the fovea, but only one of the ten eyes had associated epiretinal membrane. In every eye, the MNV was located subfoveal in the subretinal space (type 2). Seven of the ten eyes that presented without CPHF at the initial visit had never been treated, while three had received anti-VEGF injections elsewhere. SRF and IRF were commonly associated with the MNV at this time. Rarely were SHRM, hard exudates, and bleeding observed in these eyes. Six of the ten (60%) eyes were treated with an intravitreal anti-VEGF molecule at this time. Four of these six (67%) eyes were treated pro-re-nata from the start, while the remaining 2 (33%) eyes were treated with a proactive monthly loading dose regimen with at least three anti-VEGF injections.

**Table 2:** MNV characteristics at the time of presentation prior to the identification of CPHF:

Variable	Value
No. of eyes (n)	10
Mean visual acuity (logMAR), Snellen equivalent	1.308 ± 0.559 (20/407)
Type 2 MNV (n, %)	10 (100)
No. of eyes where the MNV was treated previously elsewhere (n, %)	3 (30)
No. of eyes where the MNV was untreated (n, %)	7 (70)
Subfoveal location of MNV (n, %)	10 (100)
Absent fluid (n, %)	1 (10)
Presence of sub RPE fluid (n, %)	0 (0)
Presence of SRF (n, %)	6 (60)
Presence of IRF (n, %)	9 (90)
Presence of SHRM (n, %)	1 (10)
Presence of hard exudates (n, %)	1 (10)
Presence of hemorrhage (n, %)	1 (10)
Absence of posterior hyaloid detachment at the fovea (n, %)	10 (100)
Presence of ERM at the fovea (n, %)	1 (10)
No. of eyes treated with intravitreal anti-VEGF injections (n, %)	6 (60)
No. of eyes treated with pro-re-nata regimen (n, %)	4 (67)
No. of eyes treated with proactive monthly injection regimen (n, %)	2 (33)
Mean ± SD time interval (months) for the identification of CPHF	27.3 ± 36.56
Mean number of intravitreal anti-VEGF injections taken prior to the identification of CPHF	2.4 ± 2.63 (0–9)

Abbreviations: MNV – macular neovascularization; CPHF – central posterior hyaloid fibrosis; logMAR – logarithm of minimum angle of resolution; RPE – retinal pigment epithelium; IRF – intraretinal fluid; SRF – subretinal fluid; SHRM – subretinal hyperreflective material; ERM – epiretinal membrane; VEGF – vascular endothelial growth factor; SD – standard deviation

### Features of CPHF

Table 3 describes the characteristics of CPHF eyes. In our study, 32 eyes were found to have CPHF on OCT. In the 10 eyes where prior information was available, the mean time for the identification of CPHF was 27.3 ± 24.6 months (range: 1–98 months). At the time of CPHF identification, the average logMAR visual acuity was 1.498 ± 0.374 (20/630). All 32 eyes with CPHF had type 2 MNV subtype at the subfoveal location. 69% (n = 22) of CPHF eyes had no activity in any of the fluid compartments, including the sub-RPE, sub retinal, and intra retinal spaces. SRF and IRF were found in 6 (19%) and 10 (31%) eyes respectively, at the time of CPHF identification. Other signs of MNV activity, such as SHRM, hard exudates, and hemorrhage, were uncommon in these eyes. RPE tear was observed in 6% (n = 2) of CPHF cases. At the time of CPHF, all eyes had no posterior vitreous detachment at the fovea, and 14 (41%) eyes had an associated epiretinal membrane. Ten CPHF eyes were further treated with intravitreal anti-VEGF injections, with the number of injections ranging from one to six.

**Table 3:** Optical coherence tomography (OCT) features at the time of identification of CPHF:

Variable	Value
No. of eyes (n)	32
Type 2 MNV (n, %)	32 (100)
Mean visual acuity (logMAR), Snellen equivalent	1.498 ± 0.374 (20/630)
Subfoveal location of MNV (n, %)	32 (100)
Absent fluid (n, %)	22 (69)
Presence of SRF (n, %)	6 (19)
Presence of IRF (n, %)	10 (31)
Presence of SHRM (n, %)	0 (0)
Presence of hard exudates (n, %)	1 (3)
Presence of hemorrhage (n, %)	1 (3)
Presence of RPE tear	2 (6)
Absence of posterior hyaloid detachment at the fovea	32 (100)
Presence of ERM at the fovea	13 (41)
No. of eyes treated with intravitreal anti-VEGF injections after the identification of CPHF	10 (31)
Mean ± SD number of injections given in these eyes after the identification of CPHF (Range)	0.89 ± 1.367 (1–6)

Abbreviations: MNV – macular neovascularization; CPHF – central posterior hyaloid fibrosis; logMAR – logarithm of minimum angle of resolution; RPE – retinal pigment epithelium; IRF – intraretinal fluid; SRF – subretinal fluid; SHRM – subretinal hyperreflective material; ERM – epiretinal membrane; VEGF – vascular endothelial growth factor; IQR – inter quartile range

### Details at the last follow-up visit

**Fig. 2:**
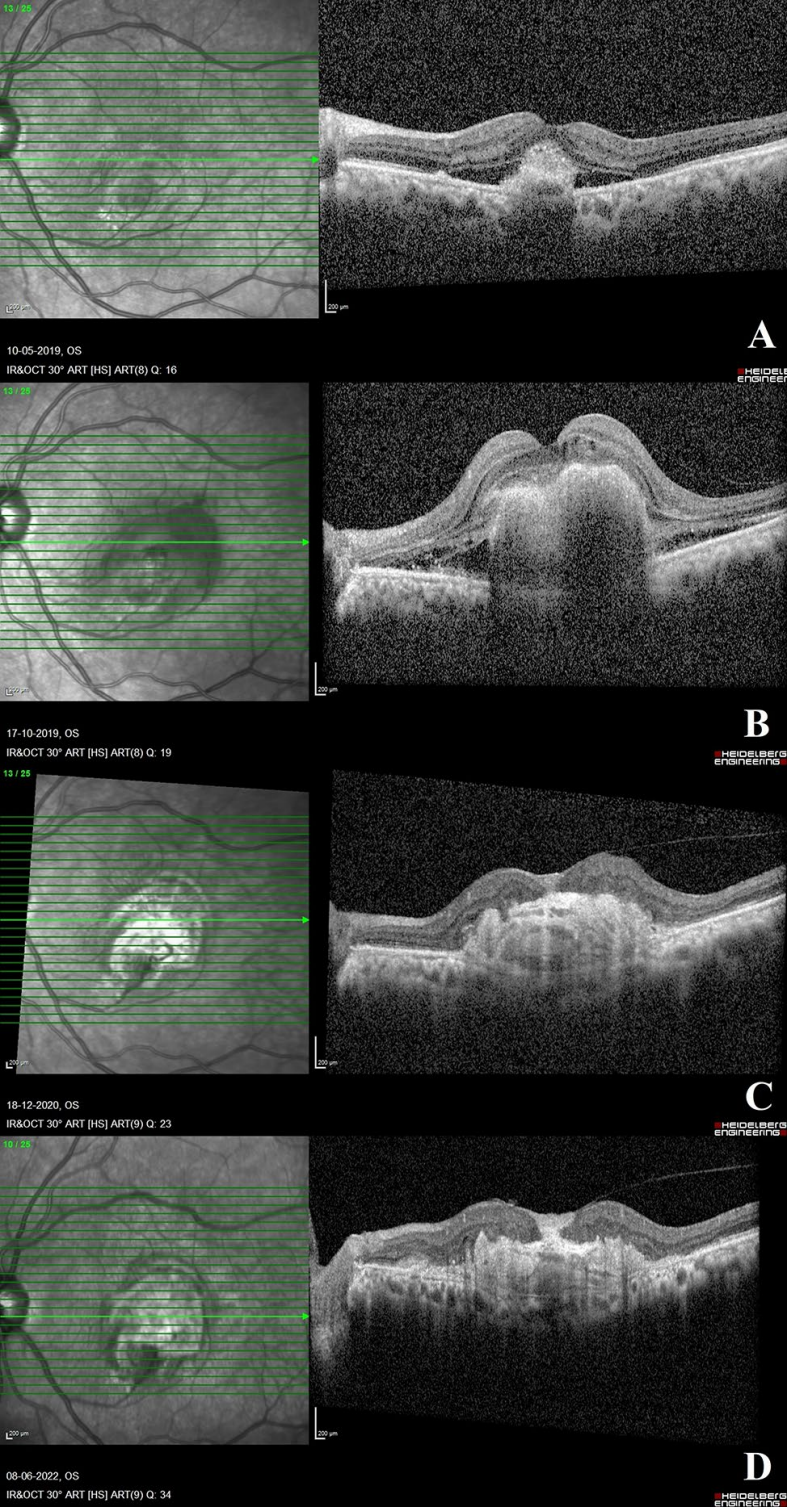
Evolution of central posterior hyaloid fibrosis (CPHF): Fig. 2**A-D**: This panel of images depicts the evolution of CPHF. At the first visit, the OCT scan shows a subfoveal macular neovascularization (MNV) of type 2 with SHRM with presence of associated subretinal fluid (Fig. 2A). This OCT scan shows no evidence of posterior vitreous separation. Visual acuity at this visit was 20/200. Evolution of CPHF: Figure 2**A** and **D** This image panel depicts the progression of central posterior hyaloid fibrosis (CPHF). At the first visit, the OCT scan reveals a subfoveal MNV of type 2 with SHRM and the presence of subretinal fluid (Fig. 2**A**) This OCT scan shows no evidence of posterior vitreous separation. Visual acuity was 20/200 at this visit. Five months after presentation, an OCT scan reveals a more fibrotic variant of subfoveal type 2 MNV with subretinal and intraretinal fluid (Fig. 2**B**). At this visit, a further decline in visual acuity to 20/400 was observed. No posterior vitreous detachment is seen. A 19-month follow-up OCT scan reveals the typical findings of CPHF (Fig. 2**C**) as described in Fig. 1. Vision is maintained at 20/400 at this time, as documented. At 18 months after the identification of CPHF at the most recent follow-up visit, the hyperreflectivity of the posterior hyaloid at the fovea has increased, while the remaining CPHF characteristics persist (Fig. 2**D**). At this visit, the visual acuity remains unchanged at 20/400. The patient received a total of two intravitreal anti-VEGF injections for that eye before the diagnosis of CPHF and one injection afterward

In 18 of the 32 eyes, further follow-up details were available. The total mean follow-up duration from CPHF identification till the last visit was 25.22 ± 24.47 months. At the last follow-up visit, the final visual acuity in these eyes was 1.304 ± 0.42 (20/402) **(**Table 4**and** Fig. 2**)**.

**Table 4:** Details of the CPHF eyes at the last follow up visit:

Variable	Value
No. of eyes with CPHF where further follow up details till the last visit were available	18
Total mean follow-up duration from CPHF identification to last visit (months)	25.22 ± 24.47
Mean visual acuity (logMAR), Snellen equivalent	1.304 ± 0.42 (20/402)

Abbreviations: CPHF – central posterior hyaloid fibrosis; logMAR – logarithm of minimum angle of resolution

### Change in visual acuity following treatment

Table 5 analyzed the visual acuity changes before and after treatment in MNV eyes before and after the development of CPHF. A significant change in visual acuity in 10 eyes prior to the CPHF development and in 18 eyes after the CPHF development following treatment was tested using the Wilcoxon matched pair sum rank test. No significant changes in visual acuity were observed at any of the study’s time points (p > 0.05).

**Table 5:** Change in the visual acuity following treatment:

	Pre-treatment logMAR visual acuity (Snellen equivalent)	Post-treatment logMAR visual acuity (Snellen equivalent)	P value
Before CPHF development (n = 10)	1.308 ± 0.559 (20/406)	1.396 ± 0.435 (20/498)	0.719
After CPHF development (n = 18)	1.370 ± 0.418 (20/469)	1.304 ± 0.42 (20/402)	0.646

Abbreviations: CPHF – central posterior hyaloid fibrosis, logMAR – logarithm of minimum angle of resolution

## Discussion

To the best of our knowledge, this is the largest CPHF series reported in the scientific literature. In this series, CPHF was a rare finding on OCT in eyes with predominantly scarred subfoveal type 2 MNVs, with or without SRF and/or IRF, and poor initial and final visual acuity. In eyes where treatment was offered, fluid resolution occurred, but there was no significant change in visual acuity during any latest follow-up visit.

We detected CPHF in 4% of eyes with all cases of nAMD and in 6% of eyes with predominantly scarred MNV, indicating that this is an uncommon finding. Khan et al. reported a series of 8 eyes in 8 patients with CPHF in advanced MNV. To calculate the prevalence of this finding, their study did not specify the exact denominator of nAMD cases and predominantly scarred MNV eyes [8].

Ten eyes of nAMD developed CPHF over time after a mean time interval of approximately 2 years from the time of initial presentation to the retina clinic. We did not find any other study to compare this information obtained from our study. Thus, we believe that the CPHF sets in after the MNV becomes chronic and predominantly scarred.

We found type 2 predominantly scarred MNV and subretinal fibrosis in all the eyes at the time of CPHF identification in our study. Even in the 10 eyes where OCT scans were available prior to the identification of CPHF, the MNV was subretinal in location i.e., type 2. The breach in the RPE also exposes the subretinal space to a variety of profibrotic factors, which may contribute to the scarring of MNV and subsequent subretinal fibrosis [10–12].

In the current series of cases, in all eyes with CPHF, a posterior cortical vitreous attachment at the fovea and a subfoveal type 2 MNV of predominantly scarred variety were identified. Thus, the fovea seemed to be the most susceptible site for the development of CPHF. There can be numerous possible explanations for why the CPHF develops at the fovea. The formation of CPHF may be explained as follows, beginning with the choroid and progressing inward towards the vitreous: (1) Existing evidence strongly suggests that inflammation plays a central role in the pathogenesis of both nAMD and non-nAMD [10, 12, 13] In MNVs, either sub RPE or subretinal, the expression of pro-inflammatory cytokines is amplified [14]. RPE performs a number of essential functions in the eye, including formation of the blood-retinal barrier, establishment of ocular immune privilege, and secretion of soluble immunomodulatory factors that mediate immunogenic inflammation [15, 16]. With a breach or tear of the RPE, abnormal choroidal vasculature grows forward in the subretinal space, while the ocular immune response is activated, producing pro-inflammatory cytokines that gain access to the subretinal and intraretinal spaces. This transretinal migration of profibrotic inflammatory cytokines may be the most likely cause for the strong adhesion of the posterior vitreous at the fovea and the development of CPHF in MNV eyes that are predominantly scarred. (2) As the subfoveal subretinal MNV advances, matures and scars, it exerts a mechanical compressive effect on the overlying fovea, rendering it the thinnest region of the macula. (3) As an advanced predominantly scarred type 2 MNV contracts, it is possible that transretinal transmission of forces originating in the subretinal space may influence the overlying attached posterior hyaloid. All of the aforementioned theories could strongly suggest that the increased adhesion of the posterior cortical vitreous at the foveal center in eyes with predominantly scarred type 2 MNV may be a consequence rather than a cause, possibly due to the release of proinflammatory/profibrotic mediators.

In this study, we did not find the presence of large RPE tears on the OCT at the time of identification of CPHF. In a previous study, our group observed large RPE tears in 33% of eyes with a bridge-arch shaped SRF and predominantly scarred MNVs on OCT [17]. This information provides two important points: (a) the absence of RPE tear in CPHF indicates the minimal vascularity and maximal scarring within the MNV complex and (b) the development of CPHF is a result of slow persistent release of the profibrotic factors caused due to the breach in the RPE which probably makes the MNV scarred and subsequently fibrotic. Also, the rarity of findings such as SHRM, hard exudates, and hemorrhage, which are all considered as indicators of disease activity, in cases of MNV with CPHF further suggested the chronic, scarred nature of the MNV complex.

The vitreomacular interface status in cases of MNV has been shown to influence the treatment response to anti-VEGF medications, primarily due to the tractional effect that would resist resolution of the underlying IRF and/or SRF [18–21]. We cannot rule out the possibility that the anti-VEGF treatment regimen contributed to the fibrotic progression. In the early disease stages, aggressive treatment of MNV with monthly loading dose injections could have prevented extension of the MNV complex into the subretinal space and the subsequent formation of subretinal scarring and CPHF. We believe that a non-aggressive intravitreal anti-VEGF treatment regimen and a breach in the RPE, which allowed the MNV to extend from the sub-RPE to the subretinal space, may have contributed to the development of CPHF in these eyes.

CPHF looks unique from other vitreomacular interface abnormalities when examined on OCT. The anteroposterior traction due to VMT obliterates or distorts the normal foveolar depression and subsequently leads to intraretinal cysts/schisis [22]. In contrast, cases of CPHF described in this series exhibited thickening of the posterior hyaloid at the fovea that appeared to fill-in the foveolar depression rather than obliterating it without causing macular schisis or macular detachment. Other vitreomacular interface abnormalities, including epiretinal membranes and taut posterior hyaloid membranes, typically manifest as inner retinal surface wrinkling [23] .Despite substantial thickening of the posterior hyaloid, our patients of CPHF did not demonstrate this surface wrinkling at the inner retina.

Analyses of ocular fluid cytokines have revealed the presence of inflammatory cytokines which are known to activate the myofibroblasts in eyes with chronic nAMD MNVs [24]. Hence, theoretically to prevent macular fibrosis at an early stage, intraocular steroids could be combined with anti-VEGF therapy. However, there is still insufficient evidence in the scientific literature to suggest that intraocular steroids could prevent the formation of macular scars in nAMD. A few reports in literature though have found intraocular steroids to be effective in improving the visual acuity in cases of poorly responsive nAMD when used as an adjunct to anti-VEGF therapy [25–27].

This study has several significant limitations. First, the design of the study as a retrospective one and the limited sample size are significant limitations. This is due to the rarity of this finding in MNV on OCT. Second, we did not conduct a comparative analysis with chronic scarred MNV cases devoid of CPHF. Third, different anti-VEGF agents were not used exclusively. It would have been interesting to compare the response of eyes with CPHF to different intravitreal anti-VEGF agents. Nevertheless, we believe the response would not have varied significantly. This study’s primary strength is that it provides valuable information about the evolution of CPHF. It identifies the factors that may have contributed to the development of CPHF in MNV patients with nAMD. This information is useful for conceptualizing the pathogenesis of CPHF and its effects on the final functional outcome. Studying the relationship between CPHF and anti-VEGF therapy would be an additional avenue for research. Further investigation is necessary to substantiate the proposition that CPHF exhibits an atypically rapid fibrotic progression. In instances of this nature, it would be advantageous to conduct an examination of the cytokine profile of vitreous samples and, if feasible, make a comparison of the histology of the posterior hyaloid face in the presence and absence of CPHF.

CPHF is a rare finding observed in eyes with advanced subfoveal type 2 MNVs with extensive subretinal fibrosis and is associated with poor visual outcomes. Its presence is reflective of MNV’s profibrotic effect on the posterior hyaloid.

## Data Availability

The datasets used and/or analysed during the current study are available from the corresponding author on reasonable request.

## Declarations

### Animal research

This article does not contain any studies with animals performed by any of the authors.

## Abbreviations

nAMD: neovascular age related macular degeneration
MNV: macular neovascularisation
OCT: optical coherence tomography
VMA: vitreo macular adhesion
VMT: vitreo macular traction
SRF: subretinal fluid
IRF: intraretinal fluid
CPHF: central posterior hyaloid fibrosis
RPE: retinal pigment epithelium
SHRM: subretinal hyperreflective material
VEGF: vascular endothelial growth factor
